# Prediction of Conserved Precursors of miRNAs and Their Mature Forms by Integrating Position-Specific Structural Features

**DOI:** 10.1371/journal.pone.0044314

**Published:** 2012-09-05

**Authors:** Goro Terai, Hiroaki Okida, Kiyoshi Asai, Toutai Mituyama

**Affiliations:** 1 INTEC Inc., Tokyo, Japan; 2 Computational Biology Research Center, National Institute of Advanced Industrial Science and Technology (AIST), Tokyo, Japan; 3 Department of Computational Biology, Graduate School of Frontier Sciences, University of Tokyo, Chiba, Japan; University of Iowa, United States of America

## Abstract

MicroRNA (miRNA) precursor hairpins have a unique secondary structure, nucleotide length, and nucleotide content that are in most cases evolutionarily conserved. The aim of this study was to utilize position-specific features of miRNA hairpins to improve their identification. To this end, we defined the evolutionary and structurally conserved features in each position of miRNA hairpins with heuristically derived values, which were successfully integrated using a probabilistic framework. Our method, miRRim2, can not only accurately detect miRNA hairpins, but infer the location of a mature miRNA sequence. To evaluate the accuracy of miRRim2, we designed a cross validation test in which the whole human genome was used for evaluation. miRRim2 could more accurately detect miRNA hairpins than the other computational predictions that had been performed on the human genome, and detect the position of the 5′-end of mature miRNAs with sensitivity and positive predictive value (PPV) above 0.4. To further evaluate miRRim2 on independent data, we applied it to the *Ciona intestinalis* genome. Our method detected 47 known miRNA hairpins among top 115 candidates, and pinpointed the 5′-end of mature miRNAs with sensitivity and PPV about 0.4. When our results were compared with deep-sequencing reads of small RNA libraries from *Ciona intestinalis* cells, we found several candidates in which the predicted mature miRNAs were in good accordance with deep-sequencing results.

## Background

**Figure 1:**
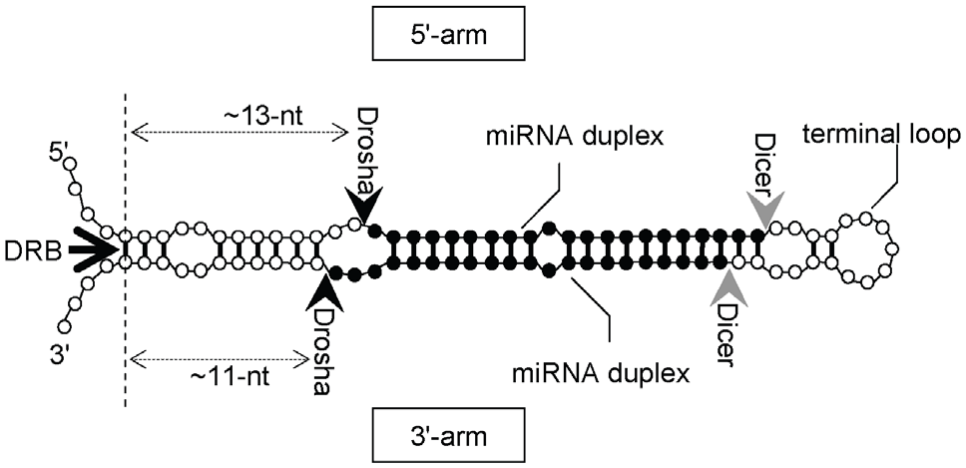
Schematic view of a putative miRNA hairpin. Cleavage sites by Drosha and Dicer are indicated by black and gray arrowheads, respectively. An miRNA duplex is represented by black circles. An arrow at the left side indicates the Drosha recognition base pair (DRB) (see text). The 5′-arm and 3′-arm indicate the 5′- and 3′-sides of a stem region in an miRNA hairpin, respectively.

MicroRNA (miRNA) is a well characterized non-coding RNA family that has important roles in various biological processes such as development [1], cancer [2], and immune response [3]. Therefore, miRNA identification and functional analysis are necessary for the understanding of many biological phenomena. An miRNA is initially transcribed as a long RNA molecule called pri-miRNA which contains one or more hairpin structures that are processed by the enzyme Drosha [4]. In this study, we refer to the hairpin structure as an “miRNA hairpin”. After an miRNA hairpin is processed into a shorter hairpin, called pre-miRNA, by Drosha, it is further processed into a ∼22-nucleotide (nt) double-stranded RNA molecule called an miRNA duplex by the enzyme Dicer [5]. Although a novel type of miRNA gene that bypasses Drosha processing has been reported [6], most miRNAs found until now are subject to Drosha processing. In general, either strand of the miRNA duplex is loaded into the RISC protein complex and functions as a mature miRNA [7]. Another strand of the miRNA duplex, which we refer to as “passenger strand”, is rapidly degraded [7]. Figure 1 illustrates the location of an miRNA duplex as well as the Drosha and Dicer cleavage sites in a putative miRNA hairpin.

Previous biochemical and computational studies have revealed several important features that are specific or necessary for miRNA hairpins. For example, miRNA duplex regions within miRNA hairpins generally form stable base pairs [8] and often have an internal small bulge in the middle [9], [10]. The 5′-end position of mature miRNA is predominantly composed of uracil and tends to be energetically unstable [9], [11]. Drosha was shown to recognize the outermost base pair in miRNA hairpins [10] and cleave the molecule at ∼13 nt and ∼11 nt from the Drosha recognition base pair (DRB; Fig. 1). Therefore, the positions around the DRB have unique secondary structural [10], [12] and evolutionary features, as shown in Results and Discussion. The length between the Drosha cleavage sites is ∼60 nt with a small variation (SD = 4.9) for human miRNA hairpins [12], although it can be longer (∼80 nt) in *Drosophila* [13].

The features described above can be useful for identifying conserved miRNA hairpins, and several methods have been proposed that take these features into account. In miRScan [14], [15], evolutionary and structural features in each part of miRNA hairpins were used for the genome-wide screening of conserved miRNA hairpins. Berezikov *et al*. [16] and RNAmicro [17] considered the degree of evolutionary conservation in a specific part of an miRNA hairpin. SSCprofiler [18] and miRRim [19] used a hidden Markov model (HMM) to model evolutionary and secondary structural features in each position of miRNA hairpins. The above methods except miRScan, however, do not explicitly take into account the location information of mature miRNAs, and therefore cannot be used to infer mature miRNAs.

More recently, several groups report new methods for detecting miRNA hairpins based on detailed structural and nucleotide features. Helvik *et al.* [20] use position-specific structural and nucleotide features for predicting the Drosha cleavage sites. They also showed that prediction of the Drosha cleavage sites improved the accuracy of detecting miRNA hairpins. MiRPred [21] detects miRNA hairpins based on ensemble of secondary structural motifs. Agrawal *et al.* [22] used context-sensitive HMM to model sequence and structure of miRNA hairpins. MiPred [23] used the Random Forest algorithm to integrate local structural characteristics and global structural stability of miRNA hairpins. Liu *et al.* [24] extracted sequence-structure motifs from miRNA hairpins and used them to distinguish true and non-miRNA hairpins. These methods, however, do not take evolutionary features into account. To use these methods for the genome-wide screening of conserved miRNA hairpins, an additional screening procedure based on the evolutionary features has to be developed, which is not a simple task because, as shown later, miRNA hairpins have a unique and complex pattern of evolutionary conservation.

In this study, we developed a new method, miRRim2, which can not only detect conserved miRNA hairpins, but also infer their mature forms. In miRRim2, each position of an miRNA hairpin is expressed as a multidimensional feature vector to detect position-specific features; therefore, an miRNA hairpin is expressed as a sequence of the feature vectors. miRNA hairpins, expressed by sequences of feature vectors, are modeled using conditional random fields (CRFs) [25], which optimize feature weights so that a trained model can most probably discriminate between miRNA hairpins and background data. The probabilistic model used in miRRim2 has several sub-components, each of which corresponds to a specific component of miRNA hairpins, such as mature miRNA, passenger strand, and terminal loop regions; therefore, the position-specific features of each component are appropriately modeled.

Recently, many miRNA hairpins have been identified that are not evolutionarily conserved. A recent study shows that the expression level of these non-conserved miRNA hairpins are very low, and that they are almost free of selective pressure [26]. Another recent study has suggested that non-conserved miRNA hairpins may disappear quickly during the course of evolution [27]. Because the biological relevance of non-conserved miRNA hairpins remains elusive, we focus on the detection of conserved miRNA hairpins, of which the biological importance is evolutionarily supported.

## Results and Discussion

### Evolutionary and Secondary Structural Features of miRNA Hairpins

**Figure 2:**
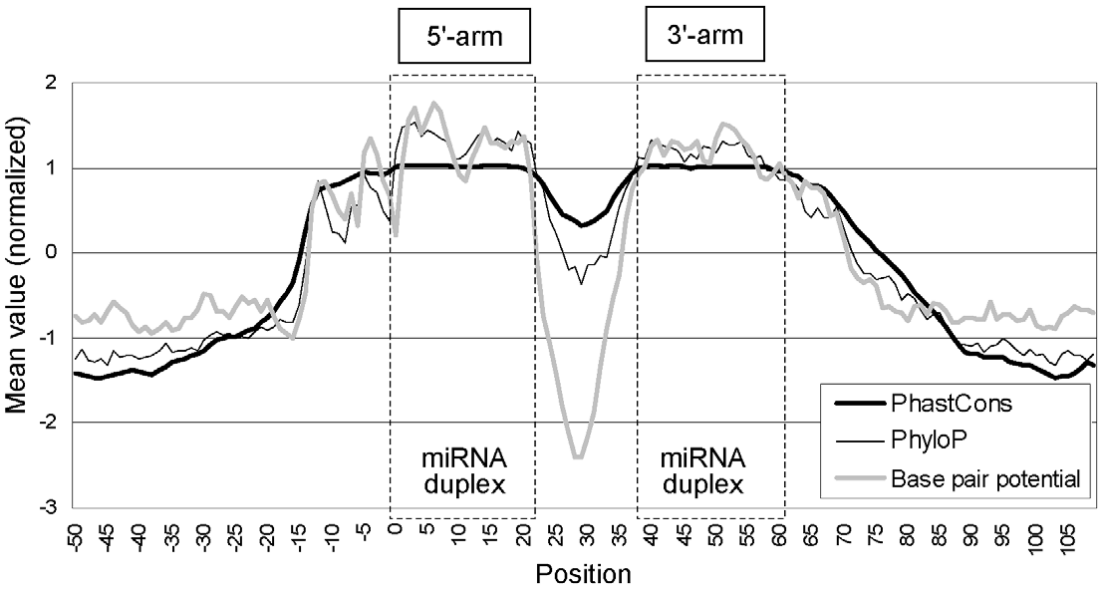
PhastCons scores, PhyloP scores, and base-pair potential averaged in each position. Position 0 indicates to 5′ ends of miRNA duplexes in the upper strand of miRNA hairpins. Dotted rectangles indicate the approximate location of the miRNA duplex.

Figure 2 shows the evolutionary and structural features of 306 conserved miRNA hairpins in human, which we refer to “core miRNA hairpins” (see Materials and Methods). In this figure, both of the PhastCons and PhyloP score represent the degree of evolutionary conservation in each position, which are calculated based on multiple alignment between species [28], [29]. The base pair potential represents the likelihood of forming a base pair in each position, which is calculated from the predicted secondary structure (see below). The position 0 in the x-axis indicates to 5′-ends of miRNA duplexes in the 5′-arm.

Overall, the PhastCons, PhyloP, and base pair potential are highly correlated, indicating that highly conserved regions tend to form base pairs in miRNA hairpins. Especially, the miRNA duplex regions are more strongly conserved and form more stable base pairs than their surrounding regions. The outside regions of the miRNA hairpin (position −20 or less, and 80 or more) are generally less conserved than the internal regions, as has been already reported [16], [19].

The Drosha recognition base pair (DRB; Fig. 1) in the 5′-arm of the miRNA hairpin is located around position -13, where the base pair potential drops sharply, as previously reported [10], [12]. Interestingly, PhastCons and the PhyloP scores also drop at the same position. The same propensity was observed around the DRB in the 3′-arm (position +11), when we adjusted position 0 to the 3′-ends of the miRNA duplex regions in the 3′-arm (Fig. S1).

**Figure 3:**
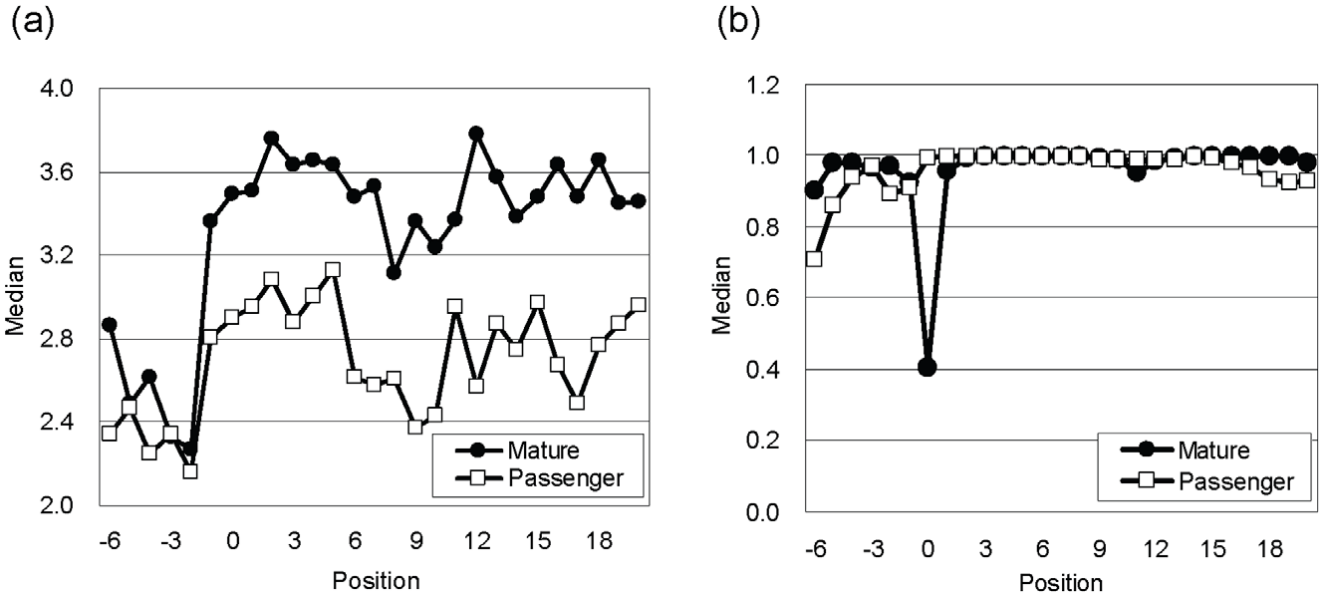
Difference between mature miRNA and passenger strand in the 5′-arm of miRNA hairpins. Median values of (a) PhyloP scores and (b) base pair potentials are plotted in each position. Position 0 indicates the 5′′-ends of mature miRNA or passenger strand.

Next we focused on the difference between the mature miRNA and passenger strand. Mature miRNAs are more strongly conserved than passenger strands (Fig. 3A), and the 5′-ends of mature miRNAs are less likely to form a base pair than passenger strands (Fig. 3B). These differences were only found for mature miRNAs in the 5′-arm. For mature miRNAs in the 3′-arm, these propensities were very weak (Fig. S2). Therefore, as shown below, the detection accuracy of mature miRNAs in the 5′-arm is higher than in the 3′-arm.

### Features used in Our Method

To utilize the evolutionary and structural features described above, we expressed each genomic position *i* as a 7-dimensional vector **o** *_i_*. Table 1 presents a brief summary of **o** *_i_*. Dimensions 1–4 represent evolutionary conservation, which are calculated from multiple alignments between species. Dimensions 5 and 6 represent secondary structural features, which are calculated from the predicted secondary structure. Dimension 7 represents a nucleotide in each position. Below, we describe the details of each dimension of **o** *_i_*.

**Table 1:** Contents of a feature vector.

Dimension	Description
1	PhastCons score
2	PhastCons score in 20-nt upstream
3	PhastCons score in 20-nt downstream
4	PhyloP score
5	Base pair potential
6	Base pair distance
7	Nucleotide

Dimension 1 of **o** *_i_* is the PhastCons score [28] of position *i*, which is calculated from multiple alignment between species. In this study, we used the PhastCons score calculated based on multiple alignment across 44 vertebrates.

Dimension 2 and 3 is the PhastCons score in position *i*-20 and *i*+20, respectively. As shown in Figure 2, the outside regions of miRNA hairpins tend to be less conserved than the internal regions, and the second and third dimensions are useful for detecting this propensity.

Dimension 4 is the PhyloP score [29] which is another measure of evolutionary conservation. The important difference between the PhyloP and PhastCons scores is that the PhyloP score is calculated independent of neighboring positions; therefore, the PhyloP score is more appropriate for evaluating the degree of evolutionary conservation at each position. In contrast, the PhastCons score is more sensitive for detecting continuous conserved regions [30].

Dimension 5 is the base pair potential which represents the likelihood of forming a base pair in each position. The base pair potential is calculated as the maximum value of base pair probabilities assigned to each position (see Methods for complete details).

Dimension 6 is the base pair distance which represents the distance between a predicted base pair. For example, if position *i* is predicted to form a base pair with position *j*, the base pair distance of position *i* is *j* – *i* (see Methods for complete details).

Dimension 7 simply represents the nucleotide (A, U, G, or C) in each position.

### The Probabilistic Model used in this Study

Because each genomic position is expressed by a 7-dimensional vector, a long genomic region is represented by a sequence of 7-dimensional vectors, and each miRNA hairpin is a sequence segment hidden in it. To detect miRNA hairpins from a long genomic region, we used a probabilistic model called a conditional random field (CRF) [25], which is recently beginning to be used in biological sequence analyses and achieves better performance than existing methods [31]–[34].

**Figure 4:**
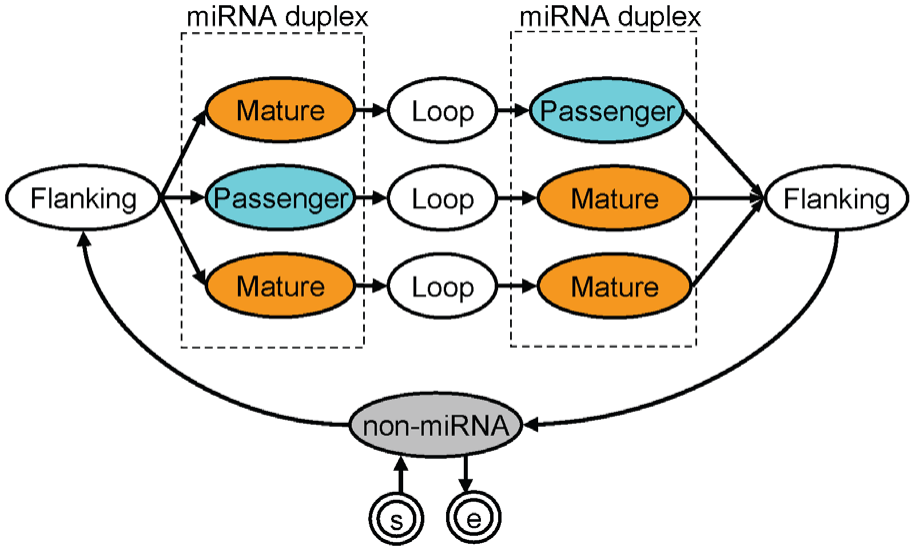
Architecture of the Model. Each sub-model is represented by an oval. The circled “s” and “e” represent a start and end state, respectively. Dotted rectangles indicate sub-models corresponding to an miRNA duplex.

The probabilistic model employed here consists of 12 sub-models (Fig. 4). The left and right sides of the Flanking sub-model represent the upstream and downstream regions of the miRNA duplex, respectively. The Loop sub-models represent the regions between miRNA duplexes. The Mature and Passenger sub-models represent the mature miRNA and passenger strand, respectively. The Non-miRNA sub-model represents regions that are not miRNA hairpins. As described above, either strand of the miRNA duplex can become mature miRNA; however, in some cases, both strands become mature miRNA. Therefore, there are 3 types of mature miRNA location in miRNA hairpins. Our architecture has 3 paths, each of which corresponds to one of the 3 types of mature miRNA location. A given sequence segment is considered an miRNA hairpin if it is derived from 1 of these 3 paths with a high probability. Similarly, a sequence segment that is expected to be derived from the Mature sub-model is considered a mature miRNA sequence.

### Scores for Detecting miRNA Hairpins and Mature miRNAs

Using the above CRF model, we calculated the probability that each genomic position *i* is an miRNA hairpin, which we denoted as P^mi^ *_i_*, using the Forward-Backward algorithm (see Methods for calculation details). We considered a continuous sequence segment of 80 base pairs (bp) or more with a P^mi^ *_i_*>*T* as a predicted miRNA hairpin, where *T* is a probabilistic threshold from 0 to 1.

We also calculated the probability of position *i* being the 5′-end position of a mature miRNA region, which we denoted as P^5end^ *_i_*. The position with P^5end^ *_i_*>*T* is considered to be the 5′-end of a mature miRNA.

### Genome-wide Cross Validation

To evaluate the accuracy of miRRim2, we designed a genome-wide cross-validation, in which the whole human genome was used for training and test data. Briefly, we selected a particular human chromosome and scanned it using miRRim2 that was trained using the core miRNA hairpins on the remaining chromosomes. To mimic a realistic situation, miRNA hairpins were excluded from training data if they were homologous to miRNA hairpins on the selected chromosome. This procedure was repeated for all chromosomes. So the whole human genome was used for evaluation. The details were described in Methods.

**Figure 5:**
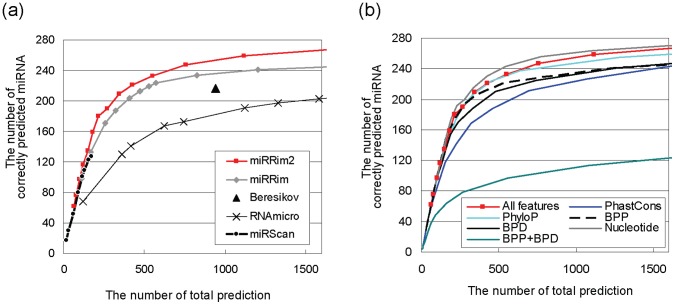
Accuracy for detecting the core miRNA hairpins. (a) The accuracy of miRRim2 together with four previously performed computational predictions is shown. (b) The change of the accuracy when one type of features is excluded. BPP: base-pair potential, BPD: base-pair distance.

The accuracy of miRRim2 is shown in Figure 5A. When the number of predicted miRNA hairpins was 216, miRRim2 could detect 180 core miRNA hairpins, indicating that miRRim2 was highly accurate at this threshold. For comparison, we obtained four publically available prediction results, and evaluated them using the same core miRNA hairpins (Figure 5A). miRRim2 could detect more core miRNA hairpins when the number of predicted miRNA hairpins was adjusted to be the same. The genomic coordinates of miRNA hairpins predicted by berezikov [16], miRscan [15], and miRRim [19] were obtained from supplemental data of these articles. Predicted miRNA hairpins of RNAmicro were obtained from the “Predicted miRNA track” of our fRNA database [35].

In order to evaluate the contribution of each type of feature to the prediction accuracy, we excluded 1 or more dimension(s) from the feature vector **o** *_i_* and investigated changes of the prediction accuracy. The result is shown in Figure 5B. The exclusion of PhastCons scores (dimensions 1–3) caused significant reduction of the prediction accuracy. The PhyloP score (dimension 4), on the other hand, had only a small effect on the prediction accuracy, indicating that only the PhastCons scores are almost sufficient for capturing the conservation pattern of miRNA hairpins.

Two types of secondary structural features (base pair potential and distance: dimensions 5 and 6) individually contribute to the prediction accuracy, although these features were dependent on each other (see Methods). When the two types of secondary structural features (dimensions 5 and 6) were simultaneously excluded, the prediction accuracy was greatly reduced, indicating that not only conservation but also secondary structural features were discriminative. The nucleotide (dimension 7) had a slightly bad effect on the prediction accuracy.

### Accuracy for Detecting the 5′-end of Mature miRNAs

**Figure 6:**
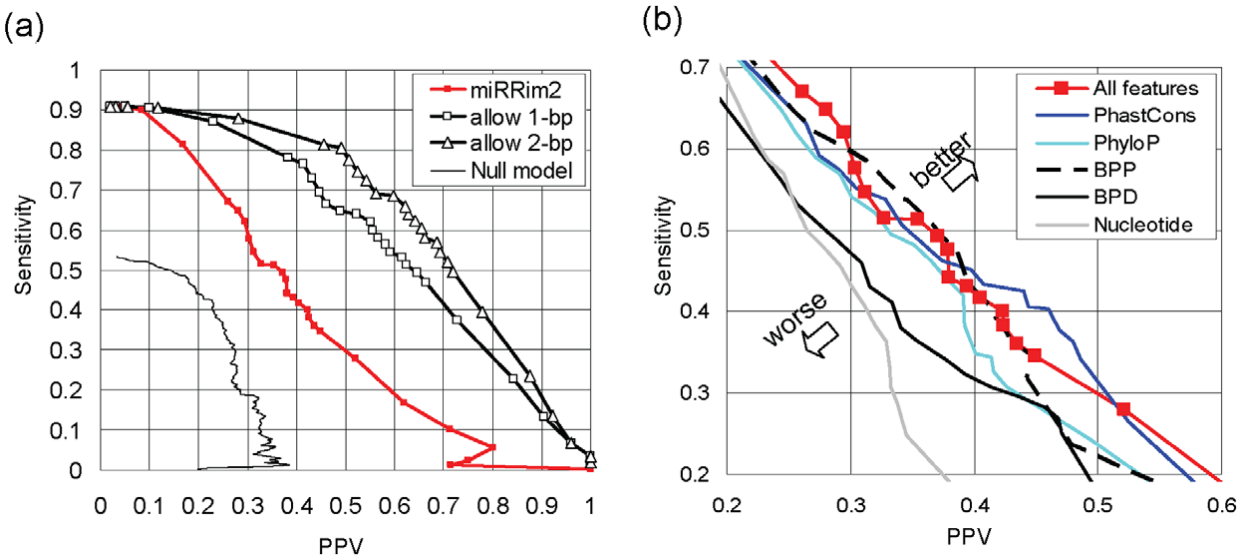
Accuracy for detecting the 5′-end of mature RNAs. (a) Sensitivity-PPV plot for mature miRNA prediction. (b) The change of the accuracy when one type of features is excluded. BPP: base-pair potential, BPD: base-pair distance.

Figure 6A shows the accuracy for detecting the 5′-end of a mature miRNA based on the cross-validation described above. Inferring the 5′-end of a mature miRNA is important because the first 8 bp from the 5′-end is so called “seed region” and plays a pivotal role in the recognition of target genes.

The prediction accuracy was measured by sensitivity and positive predictive value (PPV), which were defined as:
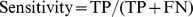


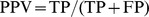
where TP, FP, and FN are the number of true positives, false positives, and false negatives, respectively. In this evaluation, the 5′-ends of true mature miRNAs within the core miRNA hairpins were defined as positive sites, and the other positions within the core miRNA hairpins were defined as negative sites. If the predicted 5′-ends were (or were not) positive sites, they were considered as true (or false) positives. Positive sites that were not detected were considered as false negatives.

Our method achieved sensitivity and PPV slightly above 0.4, which is better than our null model. In the null model, all the uracils are considered as 5¢-end of mature miRNA. Each uracil has a penalty score, which is designed such that uracils in plausible positions have low penalty score (for details, see Methods S1). When we consider the predicted sites that were 1 bp different from positive sites as true positives, sensitivity and PPV increased to about 0.55. Similarly, when we allowed a 2 bp difference, sensitivity and PPV increased to about 0.65.

Mature miRNAs in the 5′-arm of miRNA hairpins were more accurately predicted than those in the 3′-arm (Fig. S3) because the differences between mature miRNA and passenger strand are only found in the 5′-arm (see Fig. 3 and Fig. S2).

There are several methods that can identify mature miRNAs [20], [36]–[39]. Among them, only one tool MatureBayes [36] is specifically designed for predicting 5′-end of mature miRNA. In the other tools, the main purpose is to identify miRNA hairpins [37]–[39] or the Drosha cleavage sites [20], not the location of mature miRNAs. Therefore, we compared our results with MatureBayes. The prediction results of MatureBayes were obtained using the web server of MatureBayes. We used the nucleotide sequences of the core miRNA hairpins as input data of MatureBayes. The sensitivity and PPV of MatureBayes was 0.30 and 0.18, respectively. The main reason for the lower accuracy of MatureBayes than that of miRRim2 may be the difference of training data. MatureBayes was trained using both conserved and non-conserved miRNA hairpins. On the other hand, miRRim2 was trained using only conserved miRNA hairpins which were probably more uniform in terms of nucleotide contents and hairpin length than non-conserved ones.

Figure 6B shows the feature contribution to the prediction accuracy of mature miRNA. The feature that contributed the most was nucleotide (dimension 7), which may be mainly due to the fact that the 5′-ends of mature miRNAs are predominantly composed of uracil. The second most important feature was the base pair distance (dimension 6). The base pair distance was useful for identifying the approximate positions of the Drosha cleavage sites (Fig. 1) because the distance between a pair of Drosha cleavage sites in a miRNA hairpin is ∼60 bp on average with a small variation [20]. Therefore, it may also help to identify the 5′-end of a mature miRNA. The small contribution of the PhyloP score (dimension 4) may be due to the difference in evolutionary conservation between mature miRNA and passenger strand (Fig. 3A).

### Prediction of Conserved miRNA Hairpins Other than the Core miRNA Hairpins

**Figure 7:**
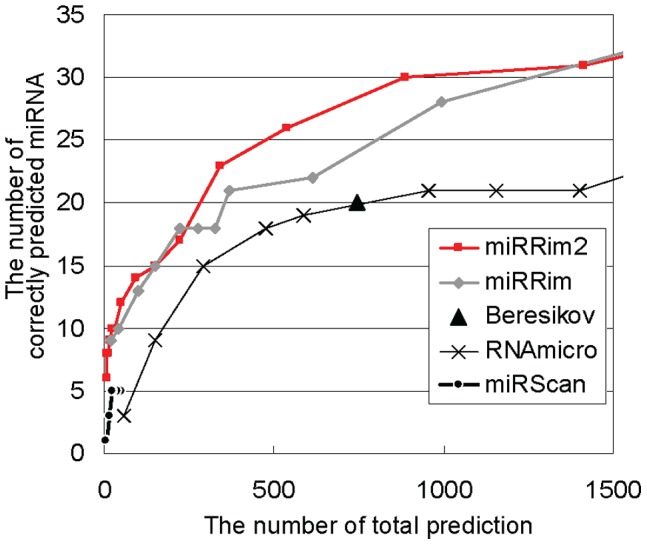
Accuracy for detecting the miRNA hairpins other than core miRNA hairpins.

The latest version of miRBase (v.18) contains 142 conserved miRNA hairpins (mean PhastCons score >0.5) that are not included in the core miRNA hairpins. About a half of them are not included because they are newly discovered after the release of miRBase version 14.0 from which the core miRNA hairpins were constructed, and another half were excluded because they were supported by only a single literature (see Methods). We investigated whether miRRim2 could detect these miRNA hairpins (Fig. 7). Several of the 142 miRNA hairpins could be accurately detected. For example, 8 of them were included in the top 11 candidates predicted by miRRim2. Overall, however, miRRim2 as well as the other four methods could not detect many of the 142 miRNA hairpins. We found that the pattern of evolutionary conservation of the 142 hairpins was considerably different from that of the core miRNA hairpins (Fig. S4). The latest version of miRBase contains many miRNA hairpins discovered by using the deep-sequencing technology. The innovation of the deep-sequencing technology may have greatly enhanced the discovery of miRNA hairpins. We, however, speculate that the deep-sequencing is too sensitive, so that it can sometimes detect miRNA-like molecules that are accidentally processed by the Drosha and Dicer. At any rate, miRRim2 seems superior to, or at lest comparable with, the other computational predictions in the detection of the 142 miRNA hairpins.

### Application to the Ciona Intestines Genome

To assess the effectiveness of miRRim2 on independent data, we applied it to the *Ciona intestinalis* genome, which was recently demonstrated to contain miRNA genes [40], [41]. For *Ciona intestinalis*, only *Ciona savignyi* is suitable for a comparative study because the other sequenced chordates are evolutionarily too distant (data not shown); therefore, the PhastCons and PhyloP scores were not available. We propose that alternative conservation scores can be calculated from pairwise genomic comparisons. As an alternative to the PhyloP score, we used the following simple score: we assigned a score = 1 to the positions with a matched nucleotide in pairwise genomic alignments, and 0 for the other positions. As an alternative to the PhastCons score, we used the alignment probability, which is a measure of the correctness of a given alignment column. Although the meaning of the alignment probability and PhastCons score is fundamentally different, they are similar in that both of them take higher values in continuous conserved segments, even if nucleotides in a certain alignment column do not coincide. Therefore, we used the alignment probability instead of the PhastCons score. Indeed, we confirmed that, even if alternative conservation scores derived from human-mouse comparison were used, we could still obtain a comparable accuracy with existing computational predictions (Fig. S5). These alternative scores were calculated using the Last program [42], which can not only perform fast genome-wide pairwise alignments, but can also calculate the alignment probability for each alignment column.

We trained miRRim2 using the core miRNA hairpins and used it to scan the *Ciona intestinalis* genome. Hendrix *et al.* [41] identified 380 miRNA hairpins in *Ciona intestinalis* using deep-sequencing experiments. In their methods, at least 5 deep-sequencing reads were needed for a certain hairpin to be considered as an miRNA hairpin. We compiled miRNA hairpins identified by Hendrix *et al.* and those included in miRBase v.18, and obtained 419 miRNA hairpins in *Ciona intesinalis*. The detection/prediction performance is shown in Figure S6A. Briefly, miRRim2 detected 47 and 73 miRNA hairpins when the number of predicted miRNA hairpins was 115 and 649, respectively. We found that the low sensitivity (73/419) was derived from the fact that only about 80 of miRNA hairpins in *Ciona intestinalis* were conserved in *Ciona savignyi* (data not shown). Because our method was designed to detect conserved miRNA hairpins, it could not detect non-conserved miRNA hairpins. In the 73 miRNA hairpins detected by miRRim2, it correctly predict the 5′-end of mature miRNA with sensitivity and PPV about 0.4 (Figure S6B).

**Figure 8:**
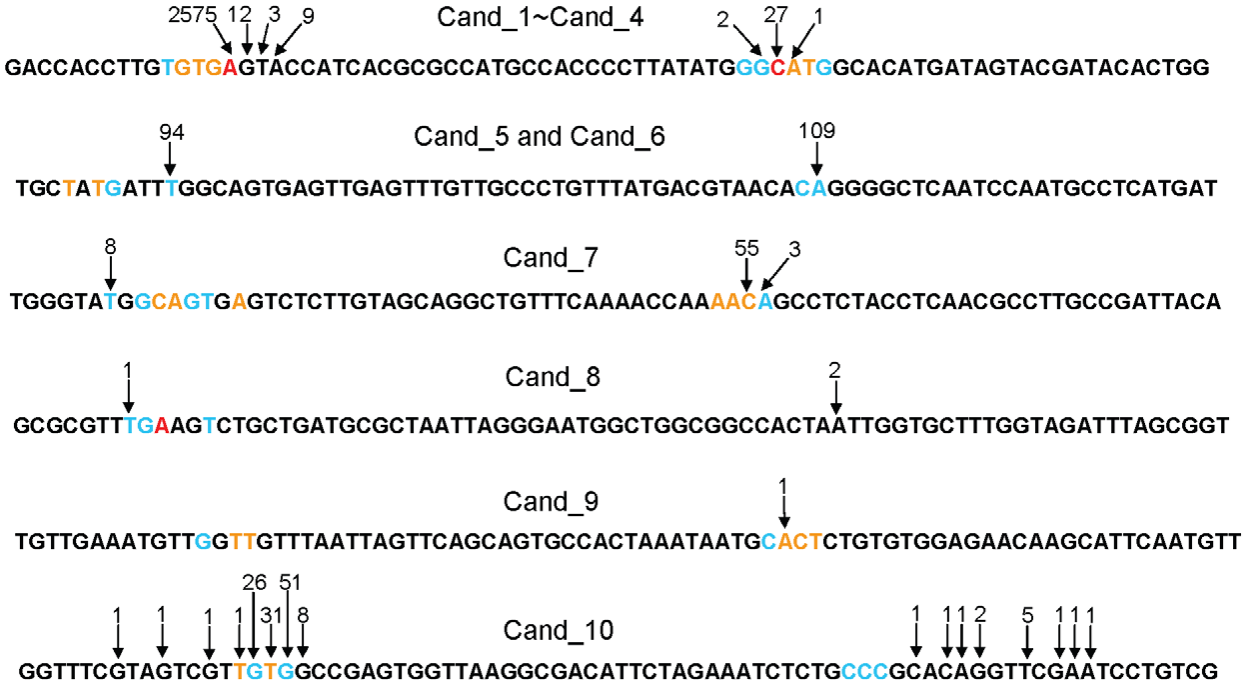
Comparison of 5 ′**-ends of mature miRNAs predicted by miRRim2 and those identified by deep-sequencing.** The probability of a predicted 5′-end (P^5end^_i_) is indicated by colours; Black, blue, orange, and red means 0≤P^5end^<0.05, 0.05≤P^5end^<0.1, 0.1≤P^5end^<0.4, and 0.4≤P^5end^, respectively. Arrows indicate the 5′-ends identified by deep-sequencing experiments by Hendrix *et al*. The number associated with an arrow indicates the number of reads.

Among the predicted miRNA hairpins, we found 10 candidates in which the locations of predicted mature miRNAs were in good agreement with deep-sequencing results. Table 2 shows the genomic coordinates of the 10 candidates. *Cand_1–Cand_4* have the same nucleotide sequence. *Cand_5* and *Cand_6* also have the same nucleotide sequence. Therefore, they may have been generated by very recent genomic duplications, although we cannot exclude the possibility that some of them are artifacts generated by the misassembly of the genomic sequence. Figure 8 shows 5¢-end positions of predicted mature miRNAs and those identified the deep-sequencing experiments by Hendrix *et al.* [41]. In many cases, predicted 5¢-ends (coloured nucleotides) are located near the 5¢-ends identified by the deep-sequencing results. *Cand_1–Cand_4* were not reported by Hendrix *et al.* [41], because they were located on recently sequenced genomic regions. *Cand_8* and *Cand_9* also were not reported by Hendrix *et al.* [41] possibly due to the small number of sequencing reads.

**Table 2:** Promising candidates.

Name	Genomic coordinatea)	Clustered miRNA
*Cand_1*	chr13p:43397-43483	cand_2
*Cand_2*	chr13p:46031-46118	cand_1
*Cand_3*	scaffold_121:338389-338465	cand_4
*Cand_4*	scaffold_121:340167-340242	cand_3
*Cand_5*	scaffold_280:34403-34489	none
*Cand_6*	chr10q:2147938-2148025	cin-mir-4054
*Cand_7*	chr10q:2150133-2150203	cin-mir-4091
*Cand_8*	chr04q:5406744-5406831	none
*Cand_9*	chr03q:5428973-5429063	cin-mir2235
*Cand_10*	chr02q:7039737-7039806	none

a) Genomic coordinate of ci2 genome (Mar. 2005 Assembly).

### Comparison of the Prediction Accuracy between Human and Ciona

miRRim2 performed better on the human genome than on the *Ciona intestinalis* genome (Figure S7 and Methods S2). Because miRRim2 uses evolutionary conservation as a key feature, its accuracy depends on the quality of evolutionary features. The higher prediction accuracy on human is due to a richer amount of comparative genomic resources for human than for Ciona.

### Difference between the Previous Version

The most important difference of miRRim2 from the previous version [19] is that it can infer the location of mature miRNAs. To our knowledge, there are no tools that can predict the 5′-end of mature miRNAs by integrating evolutionary, structural, and nucleotide features. Main methodological differences are as follows. First, a more elaborate probabilistic model was used (Fig. 4), which is designed to model each position of miRNA hairpins. In the previous version, the architecture of probabilistic model was simpler; it used an architecture consisting of linearly connected states with self-transition parameters, which was not suitable for modelling position-specific features of miRNA hairpins. Second, miRRim2 used the conditional random fields (CRFs) to train model parameters, which is recently beginning to be used in biological sequence analyses. In the previous version, hidden Markov model was used to train model parameters. Finally, in miRRim2, the base-pair distance was newly used as a feature, which significantly contributed to improve the prediction accuracy of mature miRNAs (Fig. 6B).

### Conclusions

In this study, we developed the miRRim2 method for detecting miRNA hairpins and their mature forms by integrating evolutionary, secondary structural, and nucleotide features in each position of miRNA hairpins. Our method achieved better prediction accuracy than genome-wide computational screenings previously performed by other groups. By investigating the contribution of each type of feature to the prediction accuracy, it was shown that evolutionary and secondary structural features, but not nucleotide features, are important for detecting miRNA hairpins. For the prediction accuracy of mature miRNAs, it was shown that nucleotide and secondary structural features were more important than evolutionary features. When miRRim2 was applied to the *Ciona intestinalis* genome, several promising candidates were detected. The prediction results for miRNA hairpins, miRNA duplexes, and 5′-ends of mature miRNAs in humans and *Ciona intestines* are available from http://mirrim2.ncrna.org.

## Materials and Methods

### Construction of Core miRNA Hairpins

From the 731 human miRNA hairpins in miRBase version 14.0 [43] that could be mapped on the human genome (version hg18), we selected 398 conserved miRNA hairpins with a mean PhastCons score >0.5. From these 398 miRNA hairpins, we selected 307 instances that were validated by at least two independent experimental evidences. We checked the presence of experimental evidences for each miRNA hairpin by surveying the literatures listed in miRBase version 14.0. Finally, we excluded the mirtron-type miRNA hairpins [6]. The remaining 306 miRNA were used as training and test data, which we referred to “core miRNA hairpins”. We made the length of core miRNA hairpins to be 200-bp by extending upstream and downstream regions.

For some of the core miRNA hairpins, the location of the passenger strand was not annotated. The locations of passenger strands, however, were needed for training the model parameters (see below). In such cases, they were deduced from the location of a mature miRNA assuming the 2-bp 3′-overhang illustrated in Figure 1.

### Construction of Non-miRNA Regions

Non-miRNA regions were randomly selected from non-conserved and conserved genomic regions. We selected 10000 instances from non-conserved sequence segments (mean PhastCons score <0.4) and another 10000 from conserved segments (mean PhastCons score >0.6). The length of the non-miRNA regions was 200-bp.

### Definition of a Feature Vector

In our method, each genomic position *i* was expressed by a 7-dimensional vector **o** *_i_*. We already described the complete details of dimensions 1–4 and 7 in the Result and Discussion section. Here, we described the complete details of dimensions 5 and 6.

Dimension 5 is the base pair potential which represents the likelihood of forming a base pair in each position. The base pair potential at position *i*, BPP*_i_*, is calculated as:
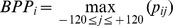
where p*_ij_* is a base pair probability between positions *i* and *j* that can be calculated by McCaskill’s algorithm [44]. We used the Rfold program (with the option L = 120) [45] for calculating base pair probabilities in a genome-wide manner.

Dimension 6 is the base pair distance which represents the distance between a predicted base pair. The base pair distance of position *i*, BPD*_i_*, is calculated by the following equation:
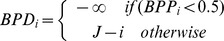
Where



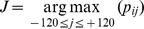



### Conversion of Continuous Values into Symbols

Dimensions 1–6 of a 7-dimensional vector **o** *_i_* are represented by continuous values. We converted them into 5 (or 6) distinct symbols, and this conversion was performed in each dimension. First, continuous values of a given dimension were obtained from the core miRNA hairpins. Continuous values belonging to the 20% or lower percentile were converted into symbol “A.” Similarly, the 20–40%, 40–60%, 60–80%, and 80% or higher percentiles were converted into “B”, “C”, “D”, and “E”, respectively. Dimension 6, which contains negative infinities, was converted into 6 distinct symbols. We converted negative infinities into symbol “F”, and the other continuous values into “A”–“E” using the same procedure as for dimensions 1–5. For dimension 7, we simply assigned “A”, “B”, “C”, and “D” to nucleotides A, U, G, and C, respectively, in order to limit the number of symbols used in our method. Therefore, the feature vector **o** *_i_* is converted to a symbol vector such as (E, B, E, E, B, C, A).

### The Architecture of Each Sub-model

The probabilistic model employed here consists of 12 sub-models (Fig. 4). The architecture of the Mature and Passenger sub-models consists of 25 connected states (Fig. S8A). Each state has an emission function, e(**o** *_i_*), that assigns a “weight” to the feature vector **o** *_i_*. As each state has its own emission function, the sub-models can capture the features in each position of mature miRNAs and passenger strands. Each connection between states has a transition parameter by which the length preference of mature miRNA regions can be modeled. For example, the propensity that mature miRNAs are 22 nt is expressed by assigning a positive large weight to the transition parameter between state 21 and 25 (the broad line in Fig. S8A). The Loop sub-model consists of 8 states, each of which is connected to itself (Fig. S8B). By using this simple architecture, we can roughly model the length and position-specific features of terminal loop regions without using a large number of states. For the Flanking sub-model, we used 20 linearly connected states (Fig. S8C) to capture the features around the DRB, which is located ∼11 or 13 nt from the Drosha cleavage sites (Fig. 1). The Non-miRNA model consists of a single state with a self transition (Fig. S8D).

### The Emission Function

Each state in our CRF model has an emission function e(**o** ***_i_***), which is defined as follows:
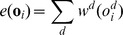
where **o** ***_i_*** is a 7-dimensional feature vector, o*_i_^d^* is a symbol in dimension *d* of **o** ***_i_***, and w*^d^*(o*_i_^d^*) is a weight assigned to the symbol o*_i_^d^* in dimension *d*. For example, the vector **o** ***_i_*** = (E, B, E, E, B, C, A) has a total weight = w^1^(E)+w^2^(B)+w^3^(E)+w^4^(E)+w^5^(B)+w^6^(C)+w^7^(A). The emission parameter, w*^d^*, was optimized from the training data.

### Training Emission and Transition Parameters

In CRFs, the conditional probability distribution P(*y*|*x*;**m**) can be directly trained from training data, where, in our case, *x* is a feature vector sequence, *y* is a sequence of “labels” assigned to *x* which reveals the location of certain sequences such as mature miRNA and the terminal loop, and **m** is a vector of emission and transition parameters. Intuitively, the parameters are optimized such that the *predicted* labels agree with the *true* labels as much as possible. This is achieved by iteratively maximizing the conditional log-likelihood of observing *true* labels.

For training the model parameters **m**, we used feature vectors corresponding to miRNA hairpins and non-miRNA regions. According to the annotation of miRBase, we assigned the labels “M”, “L”, “P”, “F”, and “N” to each position of the mature miRNA, terminal loop, passenger strand, flanking, and non-miRNA region, respectively. All the parameters **m** = (m_1_,m_2_…m*_J_*) were initially set to be 0 and were iteratively optimized using the limited-memory quasi-Newton method (L-BFGS) [46], which is a general purpose convex optimization algorithm. To prevent over-fitting, we penalized the conditional log-likelihood with the Gaussian prior 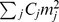. In this study, we set C*_j_* = *c* for *j* = 1, 2, …, J. The constant *c* is determined based on the prediction accuracy for a part of training data (see below).

### Determining Penalty Parameter c

In our method, the training data are divided into two groups. The first group was used to optimize transition and emission parameters, and the second one was used to determine an appropriate penalty parameter *c*. The penalty parameter is chosen from *c* = 0.1, 1, 10, 50, 100 based on the prediction accuracy for the second group. The prediction accuracy is measured based on F-score, which is a harmonic mean of sensitivity and positive predictive value (PPV). We calculate F-scores at various probabilistic thresholds, P^mi^ *_i_*, and the maximum F-score is used as a measure of the prediction accuracy.

### Genome-wide Cross Validation

We evaluated the accuracy of miRRim2 based on a genome-wide cross validation as follows. First, we selected a particular human chromosome, which we referred to as a “test chromosome”. Then, we trained miRRim2 using the core miRNA hairpins and non-miRNA regions on the remaining chromosomes, and used it to scan the test chromosome. To mimic a realistic situation, the core miRNA hairpins were excluded from training data if they were homologous to the core miRNA hairpins in the test chromosome. The information on homologues was obtained from the miFam.dat file in miRBase v.14. The training data are divided into two groups. The first group consists of randomly selected 80% of miRNA hairpins, and the same number of non-miRNA data. The second group consists of the remaining miRNA hairpins and non-miRNA data. The first group was used to optimize transition and emission parameters, and the second one was used to determine an appropriate penalty parameter *c*. This procedure was repeated for all the 24 human chromosomes. So the whole human genome was used for evaluation.

In the genome-wide cross validation, the penalty parameter *c* was determined for each of the 24 human chromosomes. For 16 of the 24 chromosomes, the prediction accuracy for the training data was highest when *c* = 10. Although we can use different *c* for each of the 24 chromosomes, we used *c* = 10 for all the 24 chromosomes.

### Definition of the miRNA Hairpin Probability and Mature miRNA Probability

To detect miRNA hairpins, we defined the probability that each genomic position *i* is an miRNA hairpin, P^mi^ *_i_*, as:
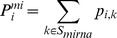
where S_mirna_ is a set of states belonging to the Flanking, Mature, Passenger, and Loop sub-models, and p*_i,k_* is a posterior probability that position *i* is derived from state *k*, which can be calculated by the Forward-Backward algorithm [47]. We considered a continuous sequence segment of 80 base pairs (bp) or more with a P^mi^ *_i_* > *T* as a predicted miRNA hairpin, where *T* is a probabilistic threshold from 0 to 1. Predicted miRNA hairpins of 150 bp or more were discarded.

The probability of position *i* being the 5′-end position of a mature miRNA region, P^5end^ *_i_*, is defined as:
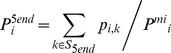
where S_5end_ is a set of the first states in all the Mature sub-models. The position with P^5end^ *_i_* > *T* is considered to be the 5′-end of a mature miRNA.

### Training of miRRim2 for the Ciona Intestinalis Genome

We used the core miRNA hairpins to train miRRim2 and used it to scan the *Ciona intestinalis* genome. The core 306 miRNA hairpins were divided into two groups. The first group consists of randomly selected 80% of miRNA hairpins, and the same number of non-miRNA data. The second group consists of the remaining miRNA hairpins and non-miRNA data. The first group was used to optimize transition and emission parameters, and the second one was used to determine an appropriate penalty parameter *c*. In this case, *c* = 10 was appropriate.

## Supporting Information

Figure S1 ***PhastCons scores, PhyloP scores, and base-pair potentialaveraged in each position.*** Position 0 indicates the 3′ ends of miRNA-duplexes in the 3′-armof miRNA hairpins. The DRB in the 3′-arm is located around position+11, where the base pair potential and PhyloP score sharply decrease.
(PDF)

Figure S2 ***Difference between mature miRNA and passenger strand in the 3***′***-arm of miRNA hairpins.*** Median values of the (a) PhyloP score and (b) base-pair potential are shown in each position. Position 0 indicates the 5′-ends of mature miRNA or passenger strands.
(PDF)

Figure S3 ***Prediction accuracy of the 5′-end of mature miRNAs.*** The detection accuracy of mature miRNAs in the 5′-arm is higher than in the 3′-arm strand.
(PDF)

Figure S4 ***The difference of a conservation pattern between the core miRNAhairpin and non-core miRNA hairpin.*** Position 0 indicates the 5′ ends of mature or passenger miRNAs in the 5′-armof miRNA hairpins.
(PDF)

Figure S5 ***Prediction accuracy of miRNA hairpins based on conservation scores calculated from Human-Mouse pair-wise alignment.***
(PDF)

Figure S6 ***The prediction performance for the Cionaintestinalisgenome.*** (a) The detection/prediction performance of miRNA hairpins. (b) Sensitivty-PPV plot for mature miRNA prediction.
(PDF)

Figure S7 ***Comparison of the prediction accuracy between human and Ciona.***
(PDF)

Figure S8 ***Architecture of each sub-model.***
(PDF)

Methods S1 ***The null model for predicting 5′-end of mature miRNAs.***
(DOC)

Methods S2 ***Comparison of the prediction accuracy between human and Ciona.***
(DOC)
