# Supplementary figures and images for: Prediction of Conserved Precursors of miRNAs and Their Mature Forms by Integrating Position-Specific Structural Features
Source: PLoS One. 2012 Sep 5;7(9):e44314. doi: 10.1371/journal.pone.0044314 (PMC3434162; doi:10.1371/journal.pone.0044314)

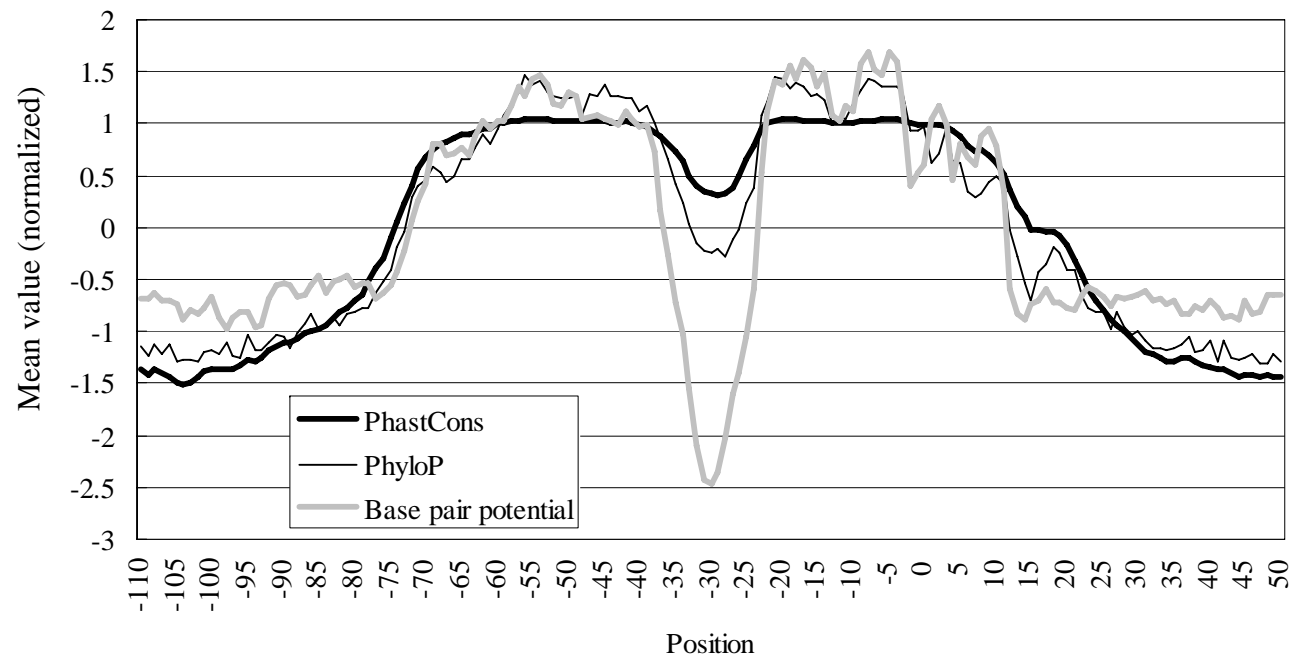

Supplement: Figure S1 — PhastCons scores, PhyloP scores, and base-pair potentialaveraged in each position. Position 0 indicates the 3′ ends of miRNA-duplexes in the 3′-armof miRNA hairpins. The DRB in the 3′-arm is located around position+11, where the base pair potential and PhyloP score sharply decrease. (PDF) [file pone.0044314.s001.pdf]

(a)

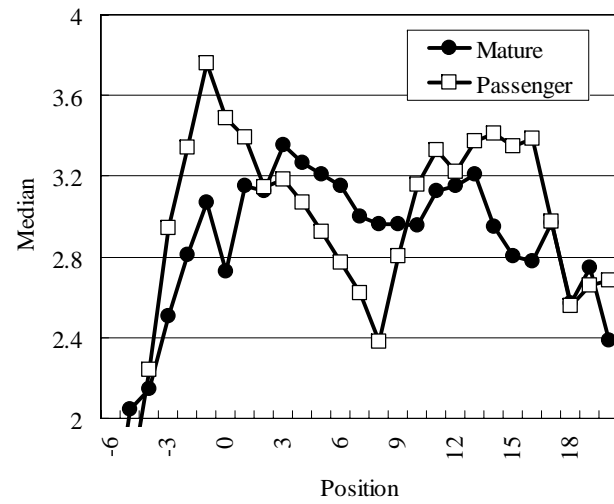

(b)

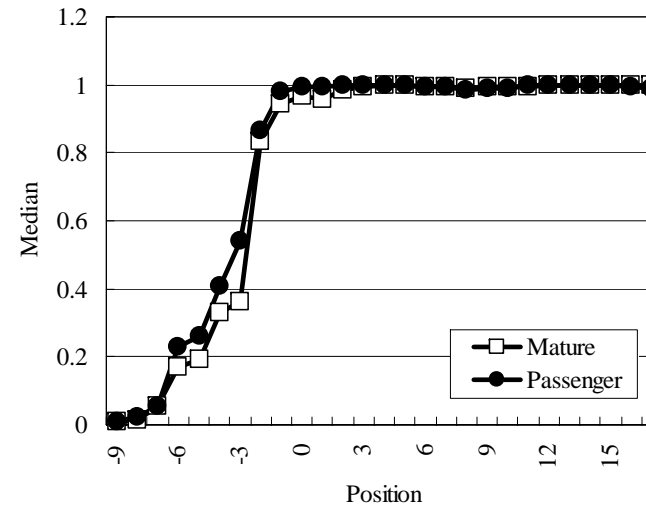

Supplement: Figure S2 — Difference between mature miRNA and passenger strand in the 3′-arm of miRNA hairpins. Median values of the (a) PhyloP score and (b) base-pair potential are shown in each position. Position 0 indicates the 5′-ends of mature miRNA or passenger strands. (PDF) [file pone.0044314.s002.pdf]

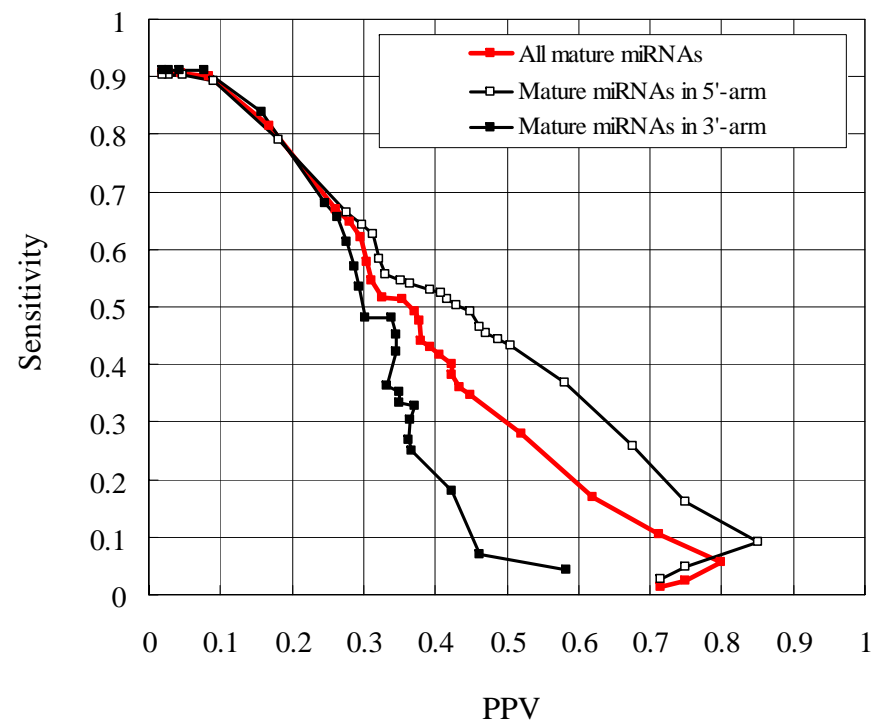

Supplement: Figure S3 — Prediction accuracy of the 5′-end of mature miRNAs. The detection accuracy of mature miRNAs in the 5′-arm is higher than in the 3′-arm strand. (PDF) [file pone.0044314.s003.pdf]

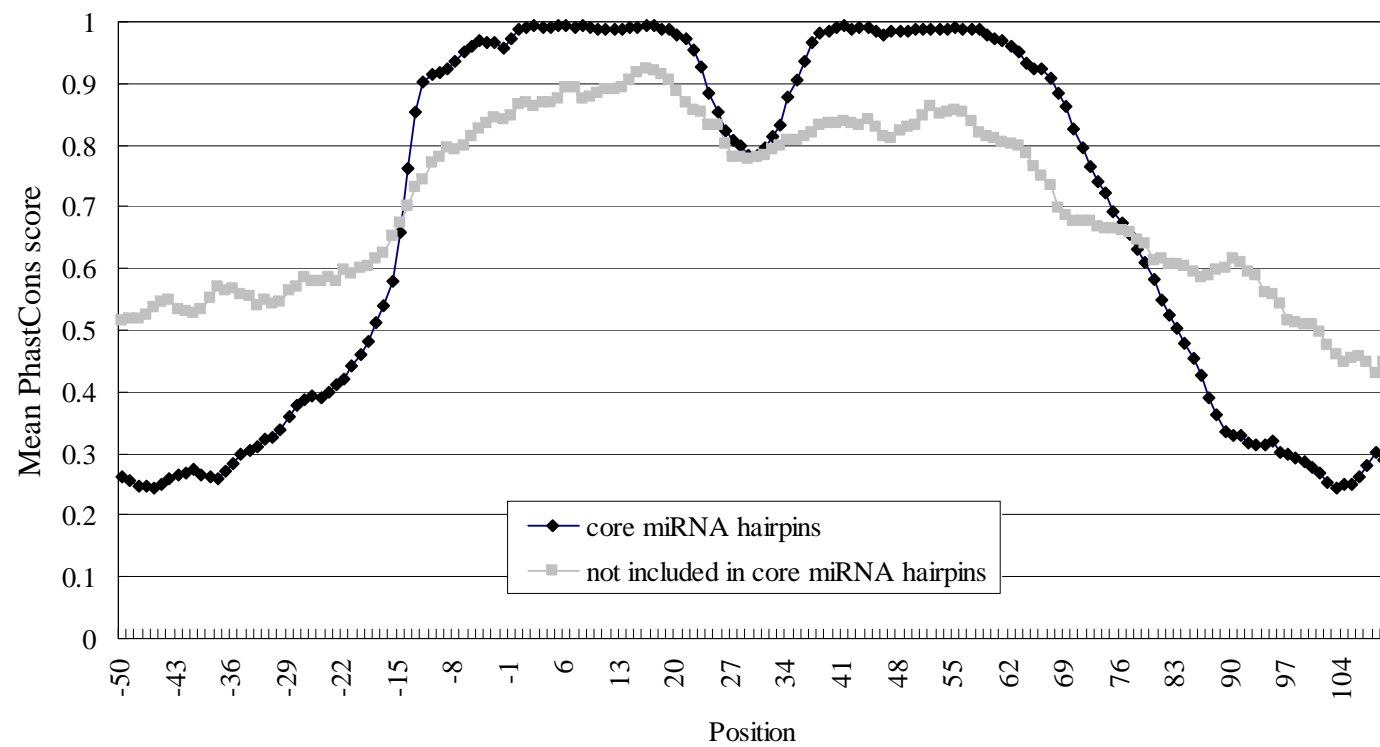

Supplement: Figure S4 — The difference of a conservation pattern between the core miRNAhairpin and non-core miRNA hairpin. Position 0 indicates the 5′ ends of mature or passenger miRNAs in the 5′-armof miRNA hairpins. (PDF) [file pone.0044314.s004.pdf]

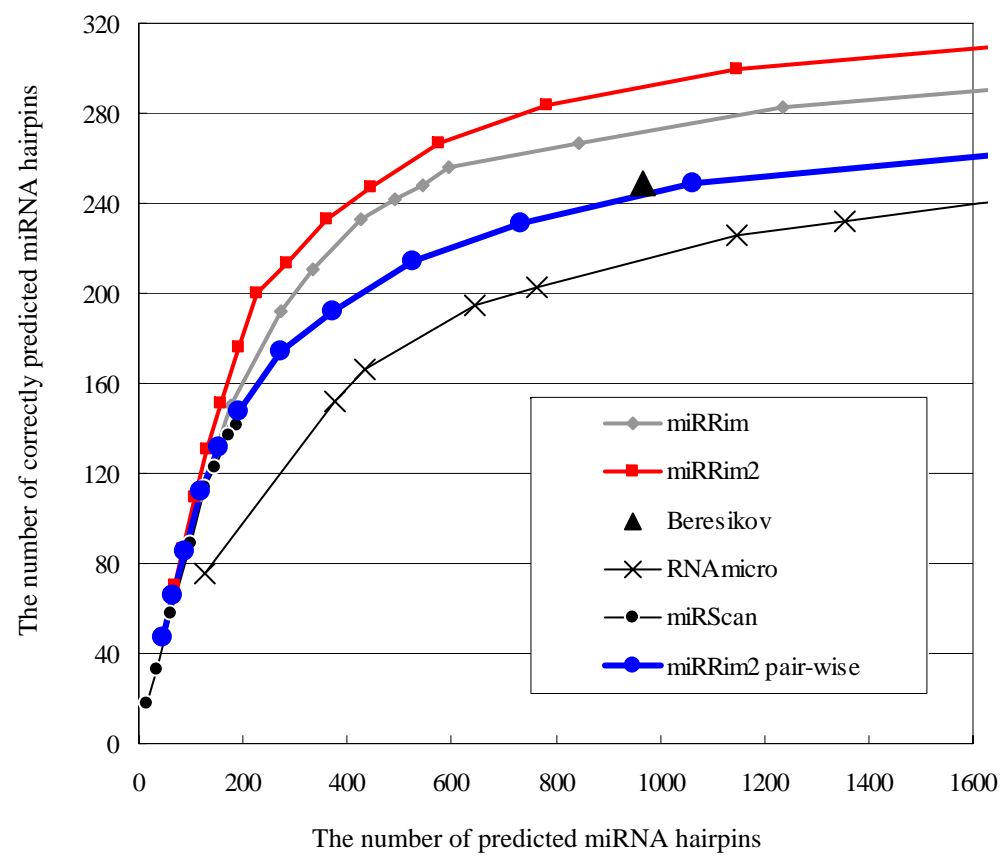

Supplement: Figure S5 — Prediction accuracy of miRNA hairpins based on conservation scores calculated from Human-Mouse pair-wise alignment. (PDF) [file pone.0044314.s005.pdf]

(a)

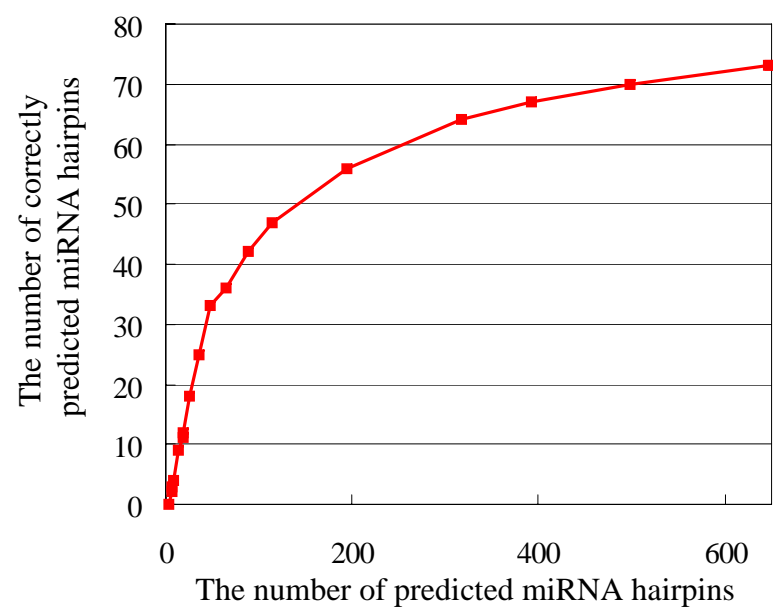

(b)

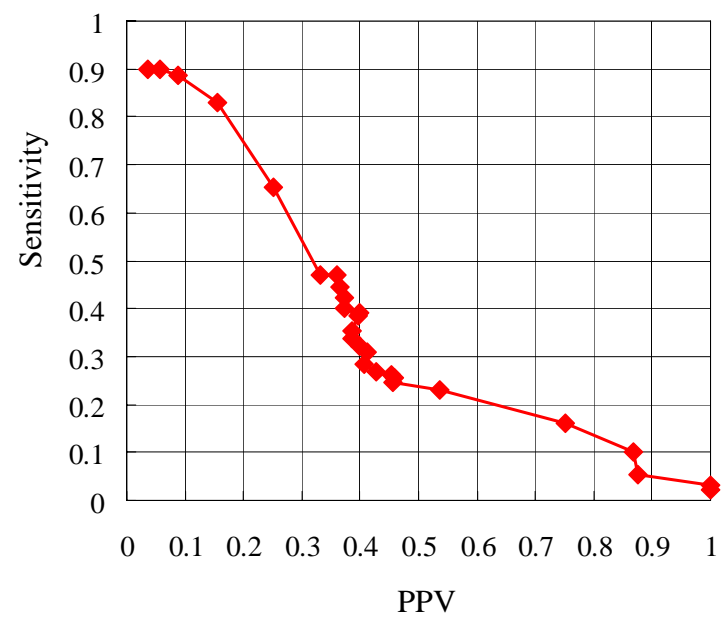

Supplement: Figure S6 — The prediction performance for the Cionaintestinalisgenome. (a) The detection/prediction performance of miRNA hairpins. (b) Sensitivty-PPV plot for mature miRNA prediction. (PDF) [file pone.0044314.s006.pdf]

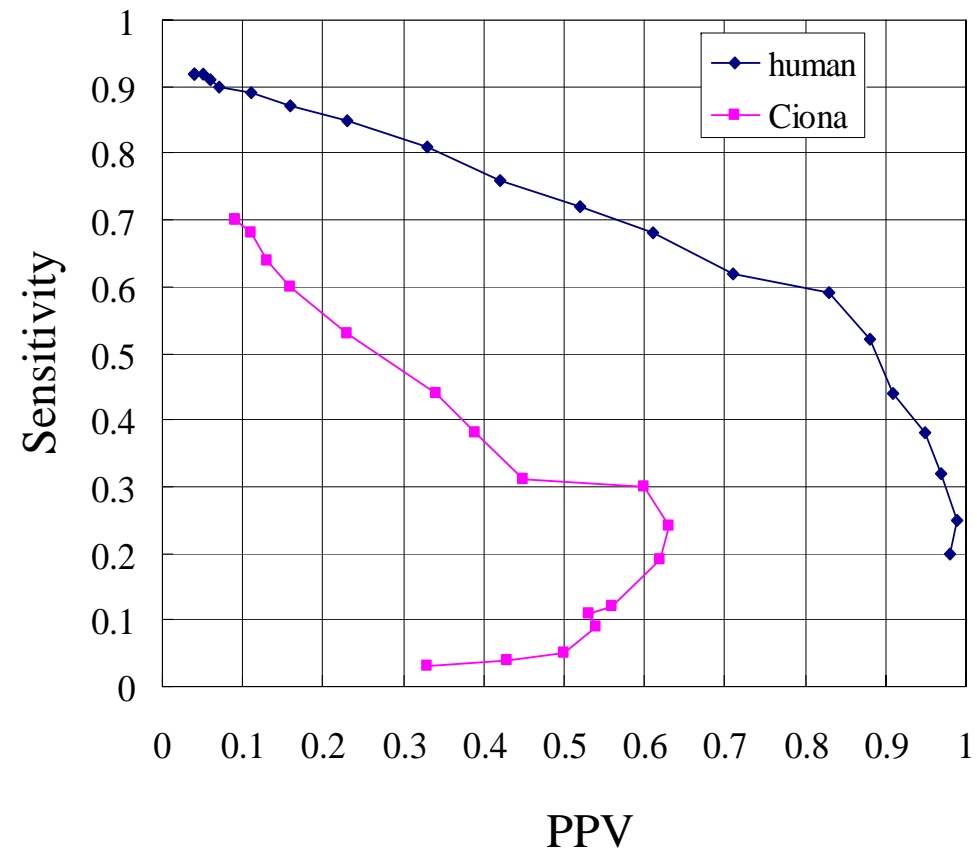

Supplement: Figure S7 — Comparison of the prediction accuracy between human and Ciona. (PDF) [file pone.0044314.s007.pdf]

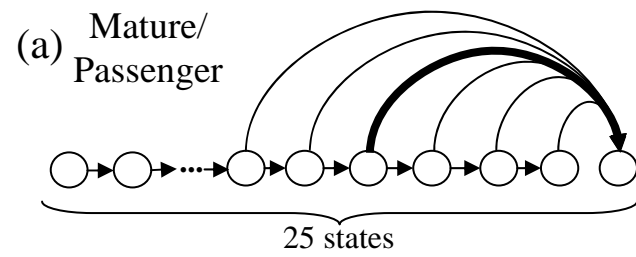

(b) Loop

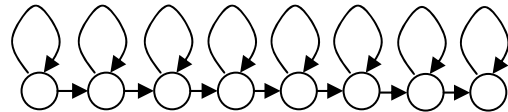

(c) Flanking

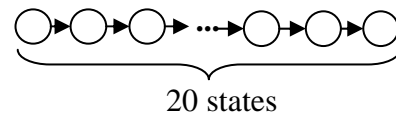

(d) Non-miRNA

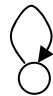

Supplement: Figure S8 — Architecture of each sub-model. (PDF) [file pone.0044314.s008.pdf]
