# Supplementary material for: Prediction of Conserved Precursors of miRNAs and Their Mature Forms by Integrating Position-Specific Structural Features
Source: PLoS One. 2012 Sep 5;7(9):e44314. doi: 10.1371/journal.pone.0044314 (PMC3434162; doi:10.1371/journal.pone.0044314)
Supplement: Methods S1 — The null model for predicting 5′-end of mature miRNAs. (DOC) [file pone.0044314.s009.doc]

The null model for predicting 5'-end of mature miRNAs.

In our null model, all the Us are considered as 5’-end of mature miRNA. Each U has a penalty score, which is designed such that Us in a plausible position have low penalty score. The penalty is defined as P = |S - *l*|, where *l* is the length between a pair of Drosha cleavage sites, *p1* and *p2*, and S is a typical length between Drosha cleavage sites. In this study, S = 60 was used.

The *p1* and *p2* were determined based on the position of U. When a given U is located on 5'-arm (Fig. A1(a)), *p1* is the position of the U, and *p2* is deduced from predicted hairpin structures assuming the 2-bp 3′-overhang. When a given U is located on 3'-arm (see, Fig. A1(b), *p1* is a 21-bp downstream position from the position of the U. Then, *p2* is deduced from predicted hairpin structures assuming the 2-bp 3′-overhang.
