# Supplementary material for: Prediction of Conserved Precursors of miRNAs and Their Mature Forms by Integrating Position-Specific Structural Features
Source: PLoS One. 2012 Sep 5;7(9):e44314. doi: 10.1371/journal.pone.0044314 (PMC3434162; doi:10.1371/journal.pone.0044314)
Supplement: Methods S2 — Comparison of the prediction accuracy between human and Ciona. (DOC) [file pone.0044314.s010.doc]

Comparison of the prediction accuracy between human and Ciona

Each species has a different number of miRNAs. Therefore, to make the comparison between species meaningful, we should use sensitivity as a measure, which is defined by TP/N, where TP is the number of true positives, and N is the number of all miRNAs. To calculate sensitivity, we need to define the dataset of all miRNAs in each species, which we denote by **A**. Because our method focused on conserved miRNAs, we assume that, in human, **A** is the “core” miRNAs, and, in Ciona, **A** is 80 miRNAs that are conserved between *Ciona intestinalis* and *Ciona savignyi*. The sensitivity and PPV for human and Ciona is shown in Figure S7.
